# Circadian disruption of memory consolidation in *Drosophila*

**DOI:** 10.3389/fnsys.2023.1129152

**Published:** 2023-03-22

**Authors:** Jerry C. P. Yin, Ethan Cui, Paul E. Hardin, Hong Zhou

**Affiliations:** ^1^Laboratory of Genetics, School of Medicine and Public Health, University of Wisconsin—Madison, Madison, WI, United States; ^2^Neurology Department, School of Medicine and Public Health, University of Wisconsin—Madison, Madison, WI, United States; ^3^Department of Biology and Center for Biological Clocks Research, Texas A&M University, College Station, College Station, TX, United States

**Keywords:** memory formation, circadian, sleep, light/dark, *Drosophila*

## Abstract

The role of the circadian system in memory formation is an important question in neurobiology. Despite this hypothesis being intuitively appealing, the existing data is confusing. Recent work in *Drosophila* has helped to clarify certain aspects of the problem, but the emerging sense is that the likely mechanisms are more complex than originally conceptualized. In this report, we identify a post-training window of time (during consolidation) when the circadian clock and its components are involved in memory formation. In the broader context, our data suggest that circadian biology might have multiple roles during memory formation. Testing for its roles at multiple timepoints, and in different cells, will be necessary to resolve some of the conflicting data.

## Introduction

Circadian rhythms influence almost all aspects of biology, from complex organismal physiologies and behaviors to cellular processes and synchronized patterns of molecular gene expression in health and disease (Chaix et al., 2016; Lee et al., 2021). The elucidation of the genes, logic, and feedback loops that constitute most eukaryotic molecular clocks has not only extended our knowledge to the mechanistic, molecular level, but reemphasized how pervasive and conserved the functions of the clock are in biology. One outstanding problem in the field is understanding the relationship between the central clock and the different “peripheral clocks” that regulate specific physiological processes (Hardin et al., 2003; Ito and Tomioka, 2016; Sehgal, 2016; Di Cara and King-Jones, 2016; Selcho et al., 2017; Yildirim et al., 2022).

Until recently, data directly linking circadian rhythms with memory formation has been somewhat limited (Eckel-Mahan et al., 2008; Gerstner et al., 2009; Gerstner and Yin, 2010; Phan et al., 2011; Xia and Storm, 2017; Price and Obrietan, 2018; Rawashdeh et al., 2018; Snider et al., 2018), or seemingly contradictory (Price et al., 2016; Wang et al., 2021). Like other circadian-regulated processes, initial experiments showed that the “time-of-day” (TOD) of behavioral training had effects on behavioral performance, with a certain time across the 24-h being optimal (Ralph et al., 2013). Animals perform best when tested at 24-h intervals from the training time, a property termed “timestamping” (Ralph et al., 2002; Cain et al., 2004a,b, Cain et al., 2017). Long-term potentiation, a popular cellular model for memory formation, exhibits TOD properties, potentially linking timing preference to a mechanism involved in memory formation (Barnes et al., 1977; Chaudhury et al., 2005; Nakatsuka and Natsume, 2014). Importantly, core molecules involved in memory formation (in different subcellular locations including at synapses) oscillate in their amounts or activities, and these are indirectly under circadian control (Eckel-Mahan et al., 2008; Phan et al., 2011; Bruning et al., 2019; Noya et al., 2019; Smies et al., 2022). Similar behavioral results in simpler organisms, including *Drosophila*, further reinforced the likely universality of circadian involvement in memory processes (Fernandez et al., 2003; Lyons et al., 2005; Lyons and Roman, 2008; Fropf et al., 2014; Lubinski and Page, 2016; Fropf et al., 2018). However, the actual role(s) of circadian biology in learning and memory formation have not been mechanistically defined in detail.

*Drosophila* has been used to investigate many problems in neurobiology, including the ground-breaking work that led to the elucidation of the molecular clock (Hall, 2017; Young, 2018; Rosbash, 2021). Two different behavioral assays have been utilized to study learning and memory formation. The olfactory avoidance behavior uses classical conditioning to test the ability of flies to associate and remember one of two odors that is presented with an electric foot shock (Tully and Quinn, 1985; Tully et al., 1994). The courtship suppression assay tests the ability of male flies to learn and remember the non-responsive mating behavior of previously mated females (Siegel and Hall, 1979). Subsequent suppression of the normally aggressive male mating behavior is used to assay learning and memory formation. The two assays differ in their ease to setup, the ability to control the salient behavioral cues, and the detailed anatomical, kinetic, and molecular information that is required during memory formation.

Sakai et al. (2004) initiated experiments showing circadian influences on courtship suppression behavior. All the circadian mutants that were tested, with the exception of *period*, did not affect the memory of courtship suppression. However, in their ground-breaking work, Inami et al. discovered that post-training incubation of flies in constant darkness (DD) but not constant light (LL), disrupted memory formation (Inami et al., 2020). They went on to define a window of time when DD incubation inhibited memory, and showed that activity of the central clock neurons, in particular the ones that secrete the peptide PDF, are required. Secretion of PDF peptides from the l-LNvs is sufficient to rescue the requirement for light, suggesting that light reception “upstream” or in these neurons results in PDF secretion. Finally, they showed that the PDFR is required and that dCREB2 activity downstream of PDF/PDFR is involved in memory formation. In a follow-up article, Inami et al. inactivated the central clock neurons prior to training and showed that this inhibited this process (Inami et al., 2021).

Lyons and Roman (2008) pioneered the demonstration that circadian biology affects fly learning in the olfactory avoidance assay. Learning is traditionally defined as immediate performance after flies are exposed to a single training trial. They showed that the TOD of training affects performance, with a peak in performance during the early nighttime period. Fropf et al. (2014) showed that the TOD of training also affects long-term memory, memory that results from 10 cycles of spaced training which is typically measured a day or more after the end of training. 1-day (1d) memory showed a TOD effect, and the core circadian gene, *period*, is somehow involved in this process (Fropf et al., 2018). A TOD effect that requires an intact central clock has also been demonstrated for the memory of a fly appetitive behavioral paradigm (Chouhan et al., 2015, 2017).

Several outstanding issues remain after the publication of these important articles. There is confusion about whether the circadian genes themselves are involved, and if so, why mutations in multiple core clock genes (*tim^01^*, *clk^jrk^*, *cyc^o^*) do not affect memory formation in courtship suppression (Sakai et al., 2004). These mutations result in “loss-of-function” phenotypes in locomotor activity, and the simple expectation is that they would disrupt memory formation if circadian genes are needed. In contrast, the *per* mutants have strong effects on performance in courtship suppression and overexpression can even enhance the process (Sakai et al., 2004). Using the olfactory avoidance behavior, Chen et al. (2012) previously showed that the dorsal anterior lateral neurons (DAL) are important for memory formation and that some of the circadian genes are expressed in those cells in response to training (Lin et al., 2021). However, just like with courtship suppression, 1d memory was unaffected in* tim^o^*, *clk^jrk^*, or* cyc^o^* mutants, while *per* mutants have a strong effect on 1d memory (Chen et al., 2012).

In this report, we use multiple approaches to ask about circadian clock involvement in memory formation. We use the term memory formation to describe the overall process of encoding, consolidation (both cellular and systems reorganization), memory maintenance, and retrieval (Squire and Alvarez, 1995). Until more behavioral, cellular, and molecular data is available, the distinctions between these processes are somewhat arbitrary. We think it is simplest to posit that maintenance begins after all anatomical reorganization (systems consolidation) is complete (Cervantes-Sandoval et al., 2013; Dubnau and Chiang, 2013). We present evidence for circadian dependency during a temporal window 1–3 days after spaced training. This window is significantly later than the 1-day timepoint which has historically been viewed as “long-term memory.” Our window is most likely only one of many transcriptional windows required during memory formation (Hirano et al., 2016; Chen et al., 2020; Mizuno et al., 2020; Inami et al., 2021). Though our results do not resolve all the questions in this field, they provide important kinetic information, genetic tools, and an experimental strategy to address these uncertainties in the future.

## Materials and methods

### Flies

The *w^1118^* (used here as a “wild type”) and the HS-*vri* stocks have been described and validated previously (Glossop et al., 2003; Gunawardhana and Hardin, 2017; Gunawardhana et al., 2020). The HS-*vri* transgene was backcrossed multiple times into the *w^1118^* background so that they are isogenic. The HS-*Clk^jrk^* transgenic fly was made from the previously described *CLK^jrk^* mutant fly (Allada et al., 1998; Darlington et al., 2000). A plasmid containing the fly *CLK^jrk^* mutant open reading frame was obtained and an EcoRI fragment was subcloned into the pCaSpeR-HS plasmid, and the resulting plasmid was injected into a *w^1118^* background stock to make transgenic flies (Thummel, 1996). That *w^1118^* background was used as the control in Figure 2, Expt #7. The *iso^31^* (used here as “wild type”) and *Pdp1*^3135^ mutant stocks have been described and validated previously (Zheng et al., 2009). The *Pdp1^3135^* mutation resides in the *iso^31^* background.

### Olfactory avoidance behavior

Young flies (less than 1 week of age) were used for behavior. Flies were collected and kept in groups of 100–150 individuals/vial and entrained to a 12-h lights on:12-h lights off schedule at 20°C for 3d prior to training. Training started between ZT = 14 and ZT = 16, and flies were returned to their dark period and kept at 20°C until testing. Testing always occurred around ZT = 16 to ZT = 18. For experiments involving changes in post-training lighting conditions, flies were incubated after training under standard LD conditions until constant conditions started, at which time the appropriate group was shifted to darkness or light (at 20°C). For the HS-*vri* flies that underwent post-training heat-shock, flies were placed in food-containing vials and incubated under light:dark control at 28°C for almost two full-days. For pre-training heat-shock, HS-*vri* flies were induced at 28°C for 3 h of time. For the HS-*Clk^jrk^* flies that underwent post-training induction, flies were transferred to empty food vials, and incubated at 36°C in a water bath for 30’ duration at 32 h and 56 h after the end of training, then returned to regular vials and incubated at 20°C under light:dark control until testing. For HS-*Clk^jrk^* flies that underwent pre-training induction, heat-shock (a 20°C to 36°C temperature shift for 30’) was done in a water bath, and flies were allowed to recover for 90’ prior to training.

Flies were trained in the olfactory avoidance-training paradigm developed by Tully and Quinn and modified to allow for automated training sessions (Tully and Quinn, 1985; Tully et al., 1994). A single-cycle of training consists of 90 s exposure to ambient air; 60 s of electric shock [the unconditioned stimulus which consists of 70 V pulses lasting 1.5 s and administered every 5 s (12 total)] accompanied by simultaneous exposure to 1 odor (the conditioned stimulus, CS+); 45 s of ambient air exposure to clear the first odor; 60 s of exposure to the second odor with no shock (the CS- condition), 45 s of ambient air to clear the second odor. This single training trial takes about 2.6 min. Spaced training consists of 10 single cycles separated by 15-min rest intervals. This training requires about 2 h 40 min of time. Massed training consists of 10 consecutive single cycles of training, and takes 37 min. Testing was done by placing flies in a choice point and giving them 2 min to decide between the CS+ and CS- stimuli. We used 3-octanol and 4-methylcyclohexanol as the odors. The performance index = [the number of flies making the correct choice] − [the number of flies making the incorrect choice]/total number of flies, multiplied by 100. To avoid odor-avoidance biases, we calculate the performance index of every single N by taking an average performance of two groups of flies, one trained with 3-octanol as CS+, and the other with 4-methylcyclohexanol. Flies were trained in a balanced manner, such that the sequence of shock-paired odors alternates, as well as the assignment of left vs. right arm at the choice point during testing. Data is presented as the standard error of the mean, and the Student’s *T*-test was used to evaluate statistical significance in pairwise comparisons.

### Sleep

Male flies within 3 days of eclosion were collected and entrained to a 12:12 light/dark cycle for 2 days before assaying for sleep. Individual flies were loaded into tubes under CO_2_ anesthesia and placed in the *Drosophila* Activity Monitor (DAM) System and put into an incubator maintained at 20°C under 12 h light:12 h dark cycles (Pfeiffenberger et al., 2010a). Raw activity counts were collected over a 24-h period. An Excel program was developed and used to calculate the various sleep measures and to plot the sleep profiles (Pfeiffenberger et al., 2010b; Cui personal communication). The sleep curves represent the average amount of sleep in each hour and the error bars represent the standard error of the mean. Dead flies (those with two or more consecutive days without locomotor activity) were manually eliminated. Sleep was measured from two groups of flies, one group was maintained in a 20°C incubator for the entire duration of the experiment. The second group was placed in a different incubator kept at 20°C until the time of heat-shock, when the temperature of the incubator was shifted to 28°C.

### Circadian locomotor activity

Male flies within 3 days of eclosion were collected and entrained to a 12:12 light/dark cycle at 20°C for 2 days prior to assaying for circadian activity. Individual flies were anesthetized using CO_2_, loaded into tubes, and placed in the DAM system to measure locomotor activity under 12:12 light/dark cycles at 20°C for 3 days (Pfeiffenberger et al., 2010a). The incubator containing the DAM monitors was shifted to constant darkness for 2 days, then shifted to 28°C for the remaining 4 days while maintained under constant darkness. Locomotor activity was monitored for the entire duration of the experiment, and data were analyzed using an Excel program we developed (Pfeiffenberger et al., 2010a; Cui personal communication). Table 2 compiles the average total number of beam crossings per minute (binned across hourly units) for the different flies and treatments during the entire duration of the recording period.

## Results

### Post-training dark periods (D) inhibit memory formation

**Figure 1:**
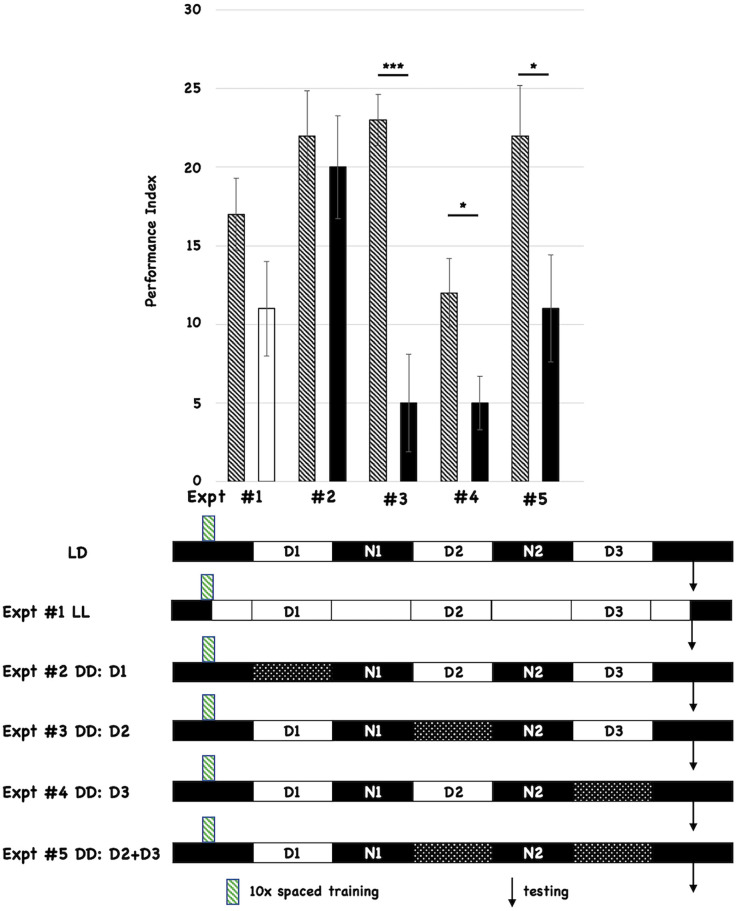
Post-training incubation of flies in dark:dark (DD) conditions disrupts 3d memory. The behavioral data and summary cartoons of the experimental timeline are presented. At the top of the Figure, the Performance Index is plotted as a function of the post-training incubation schedules, and the different pairwise comparisons are numbered (#1–5). Each experiment compares the performance of flies trained and maintained after training under control (LD) conditions (the control group) vs. an experimental group where the post-training lighting conditions change. “Wild-type” *w^1118^* flies are used for both control and experimental groups. The performance scores for the control groups are depicted using a histogram with black-and-white stripes. The performance scores of the different experimental groups are represented using a solid white (LL or constant light after training) or solid black histograms (for those that receive the imposition of one or more D periods). *N* = 8 for all experiments, *T*-tests were used to evaluate significance. *p < 0.05, ***p < 0.001. All unmarked pairwise comparisons are not significantly different. At the bottom of the Figure, the first horizontal cartoon (labeled LD) describes the standard, control experimental condition. Flies previously entrained to a 12h:12 h light:dark schedule are behaviorally trained with 10 cycles of spaced training during the early nighttime (green striped box). The different post-training periods are shown as white (12 h light) and black (12 h dark) boxes, and they are labeled (Day1 = D1, Night 1 = N1 etc.). Testing (3d after training) is denoted with the black arrow. Each subsequent horizontal timeline shows a different experiment where post-training lighting conditions are altered, and the Experiment number is indicated and corresponds to its behavioral data shown using histograms. Flies exposed to constant light from the end of training to the time of testing are shown with white boxes only. Flies that experience one or more dark periods (when light normally occurs) are shown using double cross-hatched boxes.

All flies were initially entrained to 12-h light:12-h dark cycles for 3 days prior to behavioral training (10 cycles of spaced training, indicated with the green hatched box below the histograms in Figure 1). We tested the effects of post-training changes in light:dark incubation by comparing the performance of the experimental group relative to control flies that were incubated in standard light:dark conditions after training (shown as the first cartoon at the bottom of Figure 1, and labeled LD). Each different post-training lighting condition was tested individually against the control LD group, and the behavioral data for each experiment (numbered #1-#5) is shown as a separate pair of histograms. The single hatched histogram is the control condition (post-training LD), while the white or black histograms indicate the different incubation conditions being tested. In the cartoon below the Figure, the double white bars indicate constant light, while the double-hatched bars show when D is imposed. All flies were tested for 3-day (3d) memory (indicated with black arrows on the timelines). We initially chose post-training constant light (LL; Expt #1) because it was known that this treatment disrupted the oscillations of the molecular clock (Hardin et al., 1990). However, post-training LL treatment did not significantly affect 3d memory. We then tested the effect of post-training constant darkness (DD). Imposing DD during the first light period after training (D1; Expt #2) did not affect 3d memory. However, imposing constant darkness during D2 (Expt #3), D3 (Expt #4), or both periods (D2+D3; Expt #5) disrupted 3d memory. These results almost exactly parallel those reported by Inami et al. (2020) who used the courtship suppression behavioral assay. We also tested red-eyed flies and found similar effects (see Supplementary Figure 1), suggesting that these effects are not a function of eye color or *w^1118^*-mediated retinal degeneration (Ferreiro et al., 2018).

### Post-training induction of *vrille* phenocopies D periods and specifically disrupts long-term memory

**Figure 2:**
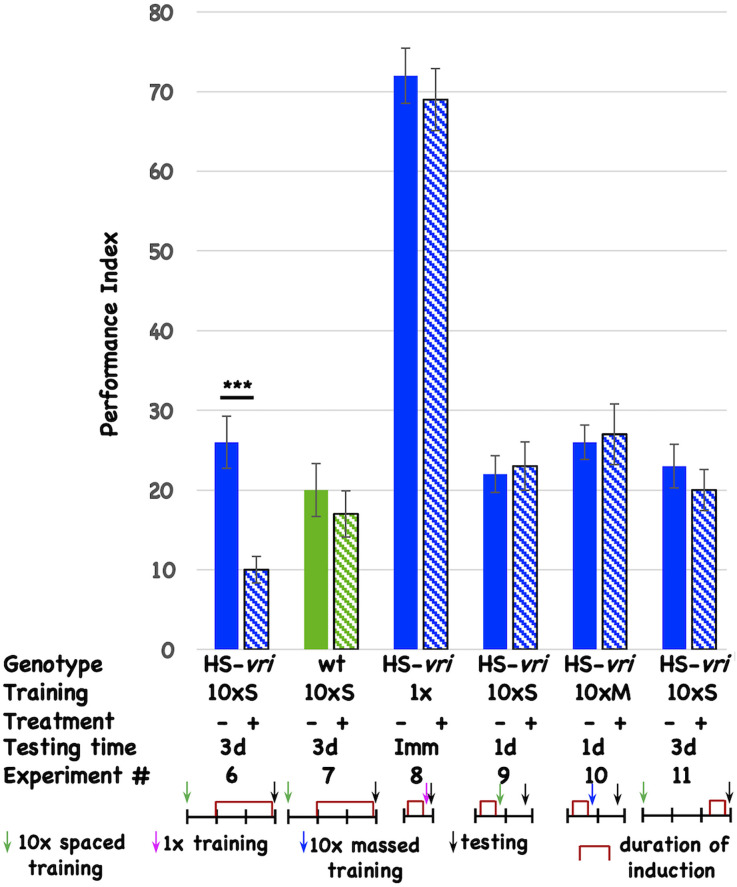
Induction of *vrille* specifically disrupts 3d memory. The performance of flies is plotted as a function of genotype, type of behavioral training, treatment, and testing time. There are two genotypes of flies: HS-*vri* transgenic (histograms in blue or blue stripes) and otherwise isogenic “wild-type” (*w^1118^*, histograms in green or green stripes) flies in which the transgene resides. The performance of flies not induced is shown with solid histograms while that of flies that undergo heat-shock induction is shown in stripes. Underneath the behavioral data, the different experiments are assigned a number, and the details of each Experiment are shown in tabular and cartoon formats. In the table, the top line indicates the genotype of the flies that are used for each experiment. 10 × S indicates flies that receive 10 cycles of spaced training. 1x denotes flies that only undergo a single training trial. 10 × M corresponds to flies that receive 10 cycles of massed training. The — sign denotes no treatment, while the + sign shows data for flies that were induced with a mild, chronic heat-shock (20°C to 28°C shift). The different testing times are denoted as 3d (3-day memory), Imm (immediate performance after a single cycle of training), or 1d (24 h memory). In the cartoon portion of the Figure, the horizontal line denotes the timeline of the experiment, and each vertical tick mark represents 1d of elapsed time. Green, magenta, and blue arrows indicate whether flies received 10 cycles of spaced training, a single cycle of training, or 10 cycles of massed training. The black arrows indicate the time of testing. The red brackets show when the heat-shock induction occurred. The color scheme used for the data histograms (top of Figure) is unrelated to the one used in the cartoons (bottom part of Figure). *N* = 8 for all experiments, *T*-tests were used to evaluate significance. ***p < 0.001. All unmarked pairwise comparisons are not significantly different.

To determine if specific circadian molecules are involved, and to minimize pleiotropic effects that circadian disruption might have on neuronal development, we use inducible transgenes to limit genetic interventions to post-development periods. The ubiquitously expressed, heat-shock-driven *vrille* transgene (HS-*vri)* has been used previously to characterize the involvement of this core circadian transcription factor in the central clock (Glossop et al., 2003). Chronic, low-level induction of the transgene beginning 1-day (1d) after the end of 10 cycles of spaced training (10 × S) affects 3d memory (Figure 2, Expt #6), while heat-shock itself does not have an effect in the isogenic wild-type stock that contains the transgene (Figure 2, Expt #7). This effect is specific for certain “phases” of memory since induction prior to 1 cycle of training does not affect immediate performance (conventionally known as learning; Figure 2, Expt #8), nor does pre-training induction affect 1d memory after 10 × S trials (Figure 2, Expt #9) or 10 cycles of massed training [Figure 2, Expt #10 conventionally known as anesthesia-resistant memory (ARM); Tully et al., 1994]. Induction of the transgene prior to behavioral testing (3d after training) likewise does not affect performance, suggesting no effects of transgene induction on retrieval (Figure 2, Expt #11).

### Disruption of other circadian genes affects long-term memory

**Figure 3:**
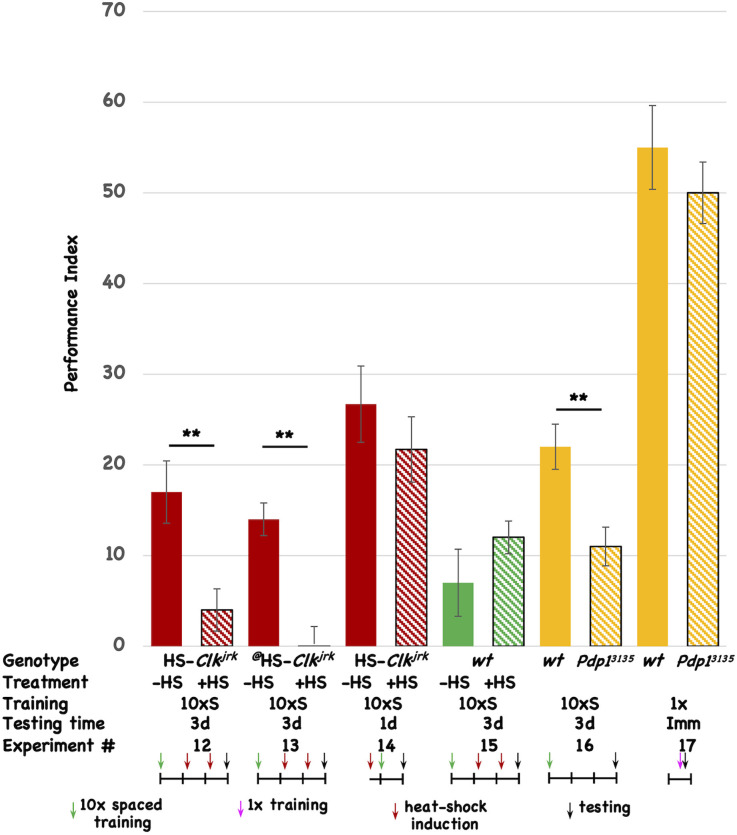
Disruption of other circadian genes also interferes with 3d memory. The performance of flies is plotted as a function of genotype, treatment, type of behavioral training, and testing time. Colored histograms (solid or striped) show the performance of HS-*Clk^jrk^* (red), *w^1118^* (green), or *iso^31^* (wt), and the *Pdp1ε^3135^* mutation (orange) that resides in the *iso^31^* background. The ^@^ symbol denotes a second, independent insertion of the HS-*Clk^jrk^* transgene. Solid colors represent the performance of uninduced (red and green) or wild type (orange; *iso^31^*) stocks. Striped histograms show the performance of the induced (red and green) or mutant lines (orange stripes). In the table, the last line is the Experiment #. The genotypes are shown in the top line, followed by whether heat-shock (+) or not was given to the flies. 10 × S corresponds to 10 cycles of spaced training, and 1x indicates flies that received only a single training trial. The testing times were immediate (Imm), 1d, or 3d after the end of training. The horizontal line in the cartoon shows the experimental timeline, and each vertical tick mark represents 1d of elapsed time. Green and magenta arrows indicate whether flies received 10 cycles of spaced training or a single cycle of training. The red arrows show the timing of a heat-shock induction (20°C to 36°C shift for 30’ duration), while the black arrows indicate the time of testing. The color scheme used for the data histograms (top of Figure) is unrelated to the one used in the cartoons (bottom part of Figure). *N* = 8 for all experiments, *T*-tests were used to evaluate significance. **p < 0.01. All unmarked pairwise comparisons are not significantly different.

To test the involvement of other circadian molecules (and the oscillatory circadian molecular network in general) we tested two other core circadian molecules for an effect on 3d memory. The *Clk* gene codes for the core transcriptional activator of E-box mediated transcription, dCLK. We made transgenic flies that express the *Clk^jrk^* ORF under the control of the heat-shock promoter. The *Clk^jrk^* mutant fly contains a truncation in the *Clk* ORF, resulting in a dominant negative protein, and heat-shock induction of this CLK^jrk^-encoding transgenic fly should disrupt the clock (Allada et al., 1998; Darlington et al., 2000). Post-training induction of two independent insertions of the *Clk^jrk^* transgene both disrupt 3d memory (Figure 3, Expt #12 and #13), but induction of one of the transgenes prior to training has no effect on 1d memory (Figure 3, Expt #14). In the wild-type fly in which the transgenes reside, post-training induction does not affect 3d memory (Figure 3, Expt #15). The induction regime that is used for these experiments involves two 30’ shifts in temperature (from 20°C to 36°C) approximately 30 and 54 h after the end of the training, with the flies returning to 20°C after the end of each heat-shock. This regimen differs from the one used for the transgenic *vri* flies (see Figure 2) and demonstrates that brief “pulses” of *Clk^jrk^* expression are sufficient to inhibit memory consolidation. We used this acute, shorter-lived induction paradigm to insure that our behavioral effects are not due to flies being exposed to a chronic heat-shock (stress) situation. Western analysis of head extracts from uninduced and induced HS-*Clk^jrk^* flies probed with Clk-specific antibody shows induction of a band of the predicted size (see Supplementary Figure 2). Three hours after induction, the transgenic CLK^jrk^ protein is in excess over the endogenous CLK protein (see Supplementary Figure 2), consistent with its seeming ability to inhibit endogenous CLK activity.

The *vri* gene encodes a D-box binding repressor, while the *Pdp1* gene makes a corresponding activator of transcription (Cyran et al., 2003; Zheng et al., 2009; Gunawardhana et al., 2020). Within the molecular circadian network, VRI and PDP1 are thought to compete for binding to D-box-containing promoters although it is not clear if they necessarily regulate the same ones. In addition to having opposite molecular functions, we chose to test *PDP1* because of the existence of the *Pdp1ε* mutation, *Pdp1*^3135^. Testing a loss-of-function mutation complements our experiments using transgenic overexpression of proteins (VRI and CLK^jrk^). The *Pdp1* gene encodes multiple protein isoforms, and Zheng et al. (2009) previously showed that *Pdp1ε* is the circadian isoform specifically disrupted in the *Pdp1^3135^* mutant. Since overexpression of VRI disrupts 3d memory, we expect that decreased PDP1 protein should do likewise. When compared to its isogenic wild-type strain, the *Pdp1^3135^* mutant shows aberrant 3d memory (Figure 3, Expt #16). To rule out a mutant effect on the relevant sensory systems involved in odor avoidance behavior, we compared the wild type and mutant stocks for immediate performance after a single training trial. Although their scores are low, there is no difference between the two stocks, suggesting that the mutant flies have comparable sensory capabilities when compared to their isogenic wild-type stock (Figure 3, Expt #17). Taken together with the *vri* data, these results suggest the involvement of *vri/Pdp1ε* in 3d memory. Four different disruptions (DD, induced overexpression of VRI, induced overexpression of CLK^jrk^, and removal of PDP1ε) all affect the same temporal window (1d-3d memory). None of the three interventions that were tested affected 1d memory. Based on this data, we believe that disruption of the circadian system very likely affects 1d-3d memory.

### HS-*vri* induction affects sleep but does not disrupt it

**Figure 4:**
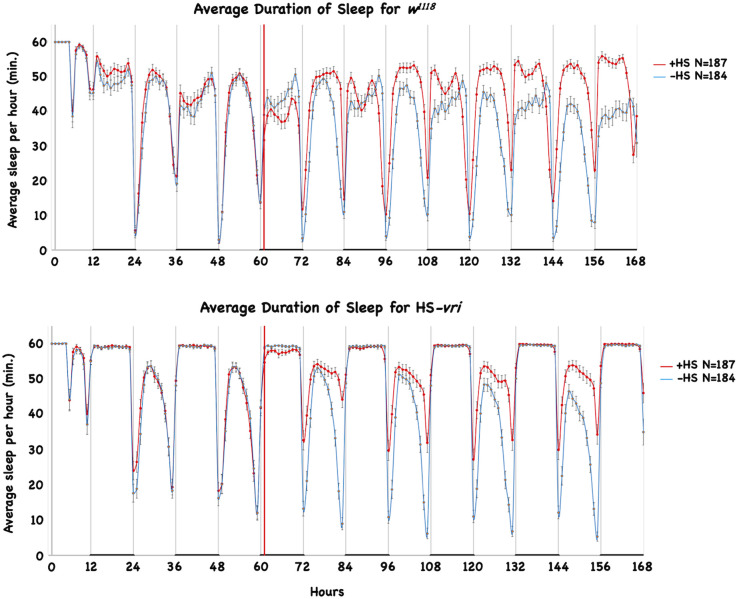
Effect of HS-*vri* induction on sleep architecture. The effect of induction on sleep architecture was measured for four different groups (*w^1118^* and HS-*vri* males, pre- and post-induction). The average minutes of sleep/hour is plotted as a function of elapsed time. For both genotypes, sleep was measured under 12-h light:12-h dark conditions, indicated with the white and black horizontal bars. Flies were entrained to a 12:12 schedule and then loaded into the DAMS monitors maintained in two different 20°C incubators with identical settings. Baseline sleep was assayed for 2 days before one of the incubators was shifted to 28°C and sleep measurements continued for flies at both temperatures. The timing of the heat-shift is shown with the dotted horizontal line. The blue and red curves represent the amounts of sleep for the non-induced and induced flies respectively.

It is generally accepted that sleep is essential for memory formation in both vertebrates and flies (Walker and Stickgold, 2004, 2006; Ganguly-Fitzgerald et al., 2006; Bushey et al., 2007; Seugnet et al., 2008; Donlea et al., 2011; Gerstner et al., 2011; Berry et al., 2015; Dissel et al., 2015; Dudai et al., 2015; Haynes et al., 2015; Liu et al., 2019; Chouhan et al., 2021). Decreases in the total amount of sleep, and/or deterioration in the quality of sleep are hypothesized to interfere with consolidation. Sleep is believed to be under homeostatic and circadian control (Borbely, 1982). Therefore we examined whether our behavioral deficits might result from disruptions to sleep. Since mild induction of the HS-*vri* transgene specifically affects 3d memory, we used the same induction protocol (20°C to 28°C chronic temperature shift) as was used for behavior to test for effects on sleep. We measured the effect of induction (Figure 4, indicated with the vertical dotted line) on sleep in the transgenic HS-*vri* male flies, as well as in isogenic control flies devoid of the transgene. Their hourly average sleep (+/–SEM) was plotted as a function of time, and Figure 4 shows that transgene induction (red trace) has small effects on the “architecture” of sleep. The transitions between active and inactive periods are identical, as are the kinetics (slopes) of “falling asleep or waking up.” Transgenic flies exposed to heat-shock sleep more during the daytime periods. As a comparison, the isogenic “wild type” stock (*w^1118^*) shows very similar effects, although these flies have less saturated sleep during both the pre- and post-induction nighttime.

The quantitative analysis of sleep is summarized in Table 1. For the HS-*vri* transgenic fly, induction increased the total amount of sleep, and this was attributable to an increase in daytime sleep. There was little effect on the total number of sleep bouts, with a small decrease in the average number of nighttime bouts that was statistically significant. In contrast, the average total length of bouts, especially those during the daytime sleep period, increased. Thus the large effects of induction were to increase the amount of sleep, and most of this occurred through increasing the average daytime length of bouts. Since transgene induction disrupted memory formation, and increasing sleep is usually thought to “enhance” memory formation, it is very unlikely that our behavioral disruption results from effects on sleep.

**Table 1:** Effect of HS-*vri* induction on sleep.

Males							
	*w^1118^*		Difference		HS-*vri*		Difference	
	-HS *N* = 187	+HS *N* = 184	%	Sig.	-HS *N* = 187	+HS *N* = 184	%	Sig.
Total sleep (min)	846	1,067	21%	^****^	1,094	1,262	13%	^****^
Day sleep (min)	371	531	30%	^****^	391	557	30%	^****^
Night sleep (min)	475	549	13%	^****^	703	687	−2%	^****^
Total bout #	31	40	23%	^****^	27	26	−4%	ns
Day bout #	17	20	15%	^****^	23	23	0%	ns
Night bout #	14	19	26%	^****^	4.1	3.4	−21%	*
Ave. bout L (min)	37	33	−12%	ns	47	67	+30%	^****^
Day bout L (min)	31	39	21%	*	20	36	+44%	^****^
Night bout L (min)	48	43	−12%	*	385	401	+4%	ns

w^1118^ and *HS-vri* flies (males) were used to assess the effect of a mild, chronic temperature shift (20°C to 28°C) on sleep. Flies loaded into the DAM apparatus were incubated in two different incubators set identically (20°C under 12:12 light:dark control). Once the flies were entrained, sleep was measured using the standard parameters for 2 days. One of the incubators was then shifted to 28°C at ZT = 12, and all groups were monitored for sleep for an additional 2 days. Each horizontal row of the Table measures a different sleep parameter: total, daytime, and nighttime sleep; total, daytime, and nighttime average number of bouts; total, daytime and nighttime average bout lengths. All values are shown in minutes. Genotypes and N size for each group are shown at the top. The Difference shows changes (positive or negative) in the induced vs. the uninduced group. The significance of the changes between the two groups is tabulated. *p < 0.05, ****p < 0.0001, ns = not significant.

For comparison, we have also measured sleep changes in the isogenic *w^1118^* strain that is the background in which the HS-*vri* transgene resides. In general, the effects of heat-shock trend in the same direction, with increases in the total amount of sleep during both daytime and nighttime periods. The average number of bouts increase (total, daytime and nighttime), while the average bout lengths (total, daytime, and nighttime) show barely significant changes. Regardless of these subtle changes, there is no measurable effect of heat-shock in *w^1118^* flies on memory formation.

### HS-*vri* induction mildly affects circadian locomotor activity

**Figure 5:**
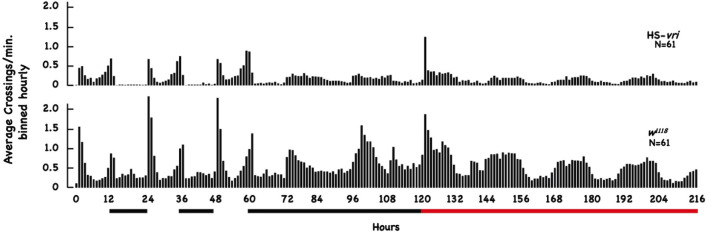
Effect of HS-*vri* induction on locomotor activity. Flies (*w^1118^* and HS-*vri*) were placed into the DAM apparatus to measure locomotor activity. The average number of beam crossings per minute binned in hourly units was calculated and plotted as a function of elapsed time. The black horizontal lines under the Figure indicate dark periods (hours 12–24 and 36–48) and constant darkness (hours 60–120). The red bar indicates constant darkness plus heat-shock (a 20°C to 28°C chronic shift beginning at the 120th h). The N sizes for each group are shown.

To evaluate the effect of our transgenic manipulations on circadian behavior, we tested the effect of HS-*vri* transgene induction on locomotor activity using the DAM apparatus across a 9-day period. Figure 5 plots the average number of beam crossings per minute binned hourly across the entire 216 h of elapsed time. This experiment involves two transitions, one from LD to DD (beginning at 60 h of elapsed time), and the other when heat-shock was begun (at 120 h elapsed time). The black bars below the plot show the nighttime periods and the beginning of constant D, and the red horizontal line indicates when chronic heat-shock (a 20°C to 28°C shift) was begun (in flies under constant darkness).

The HS-*vri* transgenic flies maintained under LD conditions show the classic “U-shaped” activity profile, with peaks at dawn and dusk. Upon the shift to DD, they almost immediately transition to a single peak in activity spread out evenly during the subjective “daytime.” Heat-shock results in an immediate spike in activity, decreases subsequent total activity slightly, but does not change the architecture (shape) of the activity profile. The *w^1118^* flies have quantitative differences in their activity (when compared to HS-*vri*) but exhibit a similar sleep profile (shape of the curve). Under LD conditions, they show a dramatic U-shaped curve, with greater total and peak activities than the HS-*vri* flies. The shift to constant darkness does not change the architecture as quickly, but by the time heat-shock is delivered, they show the inverted U-shaped, single peak that is broadly distributed across the subjective daytime. The *w^1118^* flies have a noticeably higher activity level than that of the transgenic flies. In terms of our behavioral results, the only manipulation (+HS in the HS-*vri* flies) that affects memory formation modestly decreases locomotor activity. The effect of heat-shock on locomotor activity in the *w^1118^* flies is greater but does not affect memory formation. Table 2 presents a more quantitative analysis of the locomotor activity depicted in Figure 5. The white and gray shading represent light and dark periods, while the red box indicates flies that are maintained under DD conditions but at the inducing temperature of 28°C. The top number in each box indicates the total average number of beam crossings (per minute binned in hourly units) in a 24 h period, while the second line in each box breaks this into the daytime and nighttime (shown in gray) periods. Once DD is imposed, the activity is divided between the subjective day and night periods (all shown in gray). The induced HS-*vri* flies (Days 6–8) are less active than when they were maintained under constant dark only (Days 4–5), and these flies are less active than their *w^1118^* counterparts under all conditions. Although affected, the decrease in the activity of the induced HS-*vri* flies (vs. the uninduced flies) is not a large effect, and is unlikely to contribute to their behavioral differences. We do not think the induced flies are just more “lethargic,” and less likely to move correctly at the behavioral choice point since they perform similarly to their uninduced controls after a single cycle of training (Figure 2 Expt #8), or after 10 cycles of spaced (Figure 2, Expt #9) or massed training (Figure 2, Expt #10) tested 1d after training. Their only deficit in performance is when it is measured at 3d after 10 cycles of spaced training (Figure 2, Expt #6).

**Table 2:** Effect of HS-*vri* induction on locomotor activity.

		Day 1		Day 2		Day 3		Day 4		Day 5		Day 6		Day 7		Day 8	
HS-vri		3.5		3.8		5.3		3.7		3.5		5.0		2.7		2.9	
		2.8	0.6	3.0	0.8	4.1	1.2	2.4	1.3	2.2	1.3	4.0	1.0	1.9	0.8	2.0	0.9
W^1118^		8.5		11.4		12.0		11.3		16.1		16.6		11.6		9.9	
		4.6	3.8	7.2	4.2	7.3	4.6	6.8	4.5	9.6	6.4	11.1	5.4	8.1	3.5	6.5	3.4

*The average locomotor activity across each day of an 8d experiment is presented. HS-*vri* and *w^1118^* male flies were entrained to a 12 h light:12 h dark schedule for 2 days prior to the start of the experiment. Flies were then exposed to 3 days of light:dark at 20°C, followed by 2 days of constant darkness, after which all flies were exposed to a temperature shift to 28°C. The top number in each box shows the average number of beam crossings/min averaged across 24 hourly bins, while the bottom two numbers separate that into the 12 h of day and night (Days 1–3), or the 12 h of subjective day and subjective night after constant darkness is imposed (Days 4–8). The red box around the data for Days 6–8 indicates that chronic heat-shock induction has occurred*.

## Conclusions

Our data using the imposition of post-training dark periods, inducible transgenes and a mutant provides strong evidence that circadian molecules, and probably the whole circadian system, is involved in *Drosophila* memory formation. Our effects seem specific to later memory, since immediate performance after a single training trial (learning), 1d memory after 10 spaced or 10 massed cycles of training, and retrieval are not affected. Since some, or all these different training regimens are hypothesized to affect different “phases” of memory, circadian disruption seems specific to late long-term memory (Tully et al., 1994; Yin et al., 1994, 1995). The same “circadian window” (1d-3d post-training) is affected with all our manipulations (DD, chronic or acute induction of *vri* or *Clk^jrk^*, mutant removal of *Pdp1ε*). This data, taken together with that from Inami et al. strongly argue for the importance of circadian regulation during memory formation in two different behavioral paradigms (Inami et al., 2020). In both behaviors, later long-term memory seems to be specifically affected. Our data add important kinetic and molecular information on circadian genes likely involved in this requirement. Some of our behavioral effects require heat-shock driven transgenes that may express VRI or CLK^jrk^ in cells that do not normally express these proteins. This ectopic expression could indirectly affect behavior. We think that this likelihood is low since DD and the *pdp1*^3135^ mutation have similar behavioral effects without any ectopic expression. Future experiments using more limited gene targeting will rigorously eliminate this possibility. It is also possible that the transcriptional targets whose gene expression is altered when HS-*vri (*or HS-*Clk^jrk^*) are induced and affect memory formation are outside of the circadian system. We think that this possibility is likely, but gene expression analyses in the memory cells will be needed to test this idea. The DNA sequences that CLK (E-boxes) and VRI/PDP1 (D-boxes) bind to are found broadly in promoter regions (Vinson et al., 2011; Ishibashi et al., 2019; Gunawardhana et al., 2020). Current efforts are focused on identifying when and where the circadian molecules are required.

Perhaps one of the surprising aspects of our data is that 1d memory does not seem to require the clock. Previously, both Sakai et al. and Chen et al. had reported that circadian mutants did not affect early memory formation using two different behavioral assays (Sakai et al., 2004; Chen et al., 2012). We have confirmed those findings using inducible transgenes, thus eliminating one possible source for the previously reported lack of effects. Since our transgenes affect certain memory “phases” (3d) but not others, we are confident that the reagents are effective. However, Lyons and Roman reported that learning, or immediate performance after a single training trial, is susceptible to “time-of-day” (TOD) effects (Lyons and Roman, 2008). We previously reported that the TOD also modulates 1d memory (Fropf et al., 2018). Intriguingly, the *perS* mutation, which exhibits a 19 h periodicity in locomotor behavior, may alter this preferred TOD of training (Fropf et al., 2018). These findings suggest that the circadian system, or at least some of the circadian molecules, are involved in influencing TOD preferences. It is unclear how the circadian system exerts TOD modulatory effects on learning and 1d memory, but “loss-of-function” mutations, or induced transgenes do not disrupt 1d memory. We believe that defining the anatomical and temporal requirements for the TOD preference, and 1d and 3d memory, will likely resolve some of the current contradictory results.

At face value, our finding that 3d memory is dependent upon an intact circadian system (while other phases are not) hints at significant differences between the mechanisms that support memory at those different timepoints. Since protein synthesis is likely needed for 1d memory, the subsequent requirement for circadian genes suggests a broader context in which memory consolidation occurs between 1–3d after training. This later requirement for circadian transcription is consistent with the emerging view that there are multiple “waves” of gene expression during consolidation in both flies and rodents (Hirano et al., 2016; Chen et al., 2020; Mizuno et al., 2020). These “waves” could contribute to features of memory formation, such as systems consolidation, that occur over a more prolonged period of time (Kim and Fanselow, 1992; Dudai, 2004, 2012). Our kinetic and molecular information on circadian genes that are likely involved in memory consolidation may provide important temporal windows and tools to investigate these events.

Another surprising result is that constant light (LL) does not disrupt memory consolidation, as first reported for courtship suppression memory (Inami et al., 2020). Our data completely supports the view that light itself is a necessary factor during memory formation. While LL treatment is known to interfere with the oscillations of the circadian molecular machinery, it does not affect memory formation. On the other hand, DD does not interfere with these molecular oscillations (Hardin et al., 1990; Qiu and Hardin, 1996) but disrupts memory formation. How these paradoxical findings can be resolved will likely require identification of the participating cells and an understanding of when the cells are needed during consolidation. One possible resolution is that memory formation recruits a process that is used in neural development, and this process requires light (Dapergola et al., 2021; Damulewicz et al., 2022).

How are our manipulations affecting memory consolidation? The two obvious neurobiological processes that might be targeted are sleep and circadian rhythms. However, all the measurable changes in response to HS-*vri* induction—increases in the total amounts of sleep, decreases in the average number of bouts, and increases in the average bout lengths—are usually associated with *better* sleep. Therefore, it seems highly unlikely that HS-*vri* induction is disrupting sleep and thus affecting memory consolidation. Induction of HS-*vri* has similarly small effects on the circadian regulation of locomotor activity. The overall “architecture” of activity (more activity during the subjective daytime and the least amount of activity near the end of the subjective night, and a single distributed peak in activity across a 24 h period) remains intact after heat-shock induction. The clear large effects of HS-*vri* induction on memory consolidation contrast with the minor effects on locomotor activity. We suspect that HS-*Clk^jrk^*, loss of *Pdp1ε*, and DD likewise have negligible effects on sleep and circadian locomotor activity, consistent with what Inami et al. reported for DD. It seems more likely that our effects are mediated by circadian involvement in “other” processes, perhaps ones that recruit “peripheral clocks” (Hardin et al., 2003; Ito and Tomioka, 2016; Sehgal, 2016; Di Cara and King-Jones, 2016; Selcho et al., 2017; Yildirim et al., 2022). Although our results do not clarify all the outstanding issues, they do provide an experimental template going forward to help resolve the current uncertainties.

Regardless of the mechanisms that circadian intervention affect during memory formation, our data and that of Inami et al. clearly show that light itself, and the circadian clock are required for memory formation (Inami et al., 2020, 2021). Inami et al. (2022) believe that circadian functions are involved in memory maintenance and we conceptually agree with this intuitively appealing idea. However, we believe that the “circadian window” described in this report is affecting an earlier part of memory formation. Future experiments will be needed to clarify the cells and the timepoints when maintenance occurs, and how the circadian system contributes to this process.

## Data availability statement

The original contributions presented in the study are included in the article/Supplementary materials, further inquiries can be directed to the corresponding author.

## Ethics statement

The research is conducted with *Drosophila*, and the University of Wisconsin-Madison Office of Vice Chancellor for Research and Graduate Education does not require ethical approval for work with invertebrates.

## Author contributions

JY conceptualized, designed and helped acquire the data, analyzed the data, and wrote the manuscript. EC did the sleep and circadian experiments and wrote the Excel program to analyze the sleep and circadian data. PH helped write the manuscript, supplied key reagents and consulted on experimental design. HZ did the research experiments and helped analyze the data. All authors contributed to the article and approved the submitted version.

## Supplementary material

SUPPLEMENTARY FIGURE 1 Post-training DD inhibits 3d memory in red-eyed flies. Uninduced HS-Clk^jrk^ (red-eyed) flies were entrained to a 12 h light:12 h dark schedule at 20°C. Flies were trained with 10 cycles of spaced training beginning around ZT = 14. In Experiment S1, the flies were incubated after training in light:dark until Day 2, at which time half of the flies (whose subsequent performance is shown with a black histogram) were exposed to dark during the D2 period and then returned to light:dark until they were tested at 3 days post-training. The other half of the flies remained on light:dark throughout and were also tested for 3d memory (hatched histogram). In Experiment S2, the experimental flies (black histogram) were shifted to constant darkness at the beginning of D2 and remained in darkness until testing. Control flies (hatched histogram) were put on LD after training and remained under those conditions until testing. ***p* < 0.01, ****p* < 0.001, *N* = 8, *T*-test.

SUPPLEMENTARY FIGURE 2 Western analysis of HS-*Clk^jrk^* induction. HS-*Clk^jrk^* transgenic flies were entrained to a 12 h light:12 h dark schedule at 20°C. Flies in groups of approximately 100 were induced, or not, in empty food vials placed into a water bath at 36°C for 30’ at ~ZT = 7. Uninduced flies were handled, transferred to vials, but not induced. After induction (or handling only), flies were returned to food vials and incubated at 20°C for 3 h, when they were collected and flash frozen in 15 ml polypropylene tubes. The tubes were shaken and pounded, and heads were isolated using a series of sieves. 50 heads were counted out (over powdered dry ice), pulverized using a dounce-like plastic pestle, and extracts made in standard 2× Laemmli SDS loading buffer. About 15 fly head-equivalents were loaded onto a 5% polyacrylamide gel, subjected to electrophoresis, processed for western analysis using a CLK-specific antibody (gp50; Houl et al., 2006, 2008) that was used at a 1:2,000 dilution. The secondary antibody (LI-COR) was a donkey anti-guinea pig IgG conjugated with a fluor and used at a 1:20,000 dilution. The mobility of the molecular weight markers (in kD) is indicated to the left of the image. Based on mobility, the endogenous CLK and transgenic CLK^jrk^ bands are indicated. Imaging and quantitation was done on the LI-COR Odyssey system. For quantitation, background counts (from a region of the gel that did not contain protein samples) was subtracted from both the presumed CLK^jrk^ and endogenous CLK bands. The resulting CLK^jrk^ intensity was greater than the adjusted intensity for the endogenous CLK band at the 3 h post-induction time point.
