# Supplementary figures and images for: Circadian disruption of memory consolidation in Drosophila
Source: Front Syst Neurosci. 2023 Mar 22;17:1129152. doi: 10.3389/fnsys.2023.1129152 (PMC10073699; doi:10.3389/fnsys.2023.1129152)

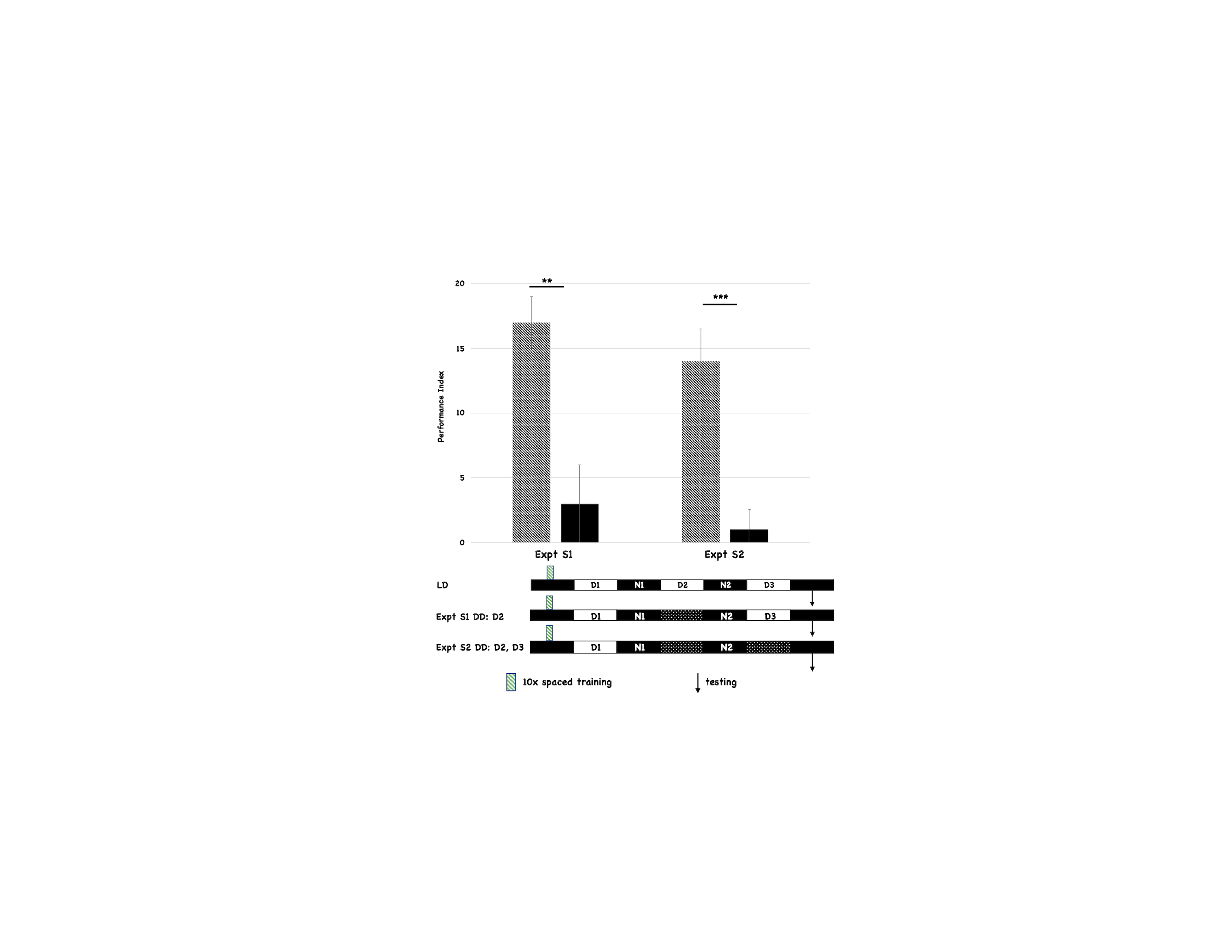

Supplement: SUPPLEMENTARY FIGURE 1 — Post-training DD inhibits 3d memory in red-eyed flies. Uninduced HS-Clkjrk (red-eyed) flies were entrained to a 12 h light:12 h dark schedule at 20°C. Flies were trained with 10 cycles of spaced training beginning around ZT = 14. In Experiment S1, the flies were incubated after training in light:dark until Day 2, at which time half of the flies (whose subsequent performance is shown with a black histogram) were exposed to dark during the D2 period and then returned to light:dark until they were tested at 3 days post-training. The other half of the flies remained on light:dark throughout and were also tested for 3d memory (hatched histogram). In Experiment S2, the experimental flies (black histogram) were shifted to constant darkness at the beginning of D2 and remained in darkness until testing. Control flies (hatched histogram) were put on LD after training and remained under those conditions until testing. **p < 0.01, ***p < 0.001, N = 8, T-test. [file Image_1.TIFF]

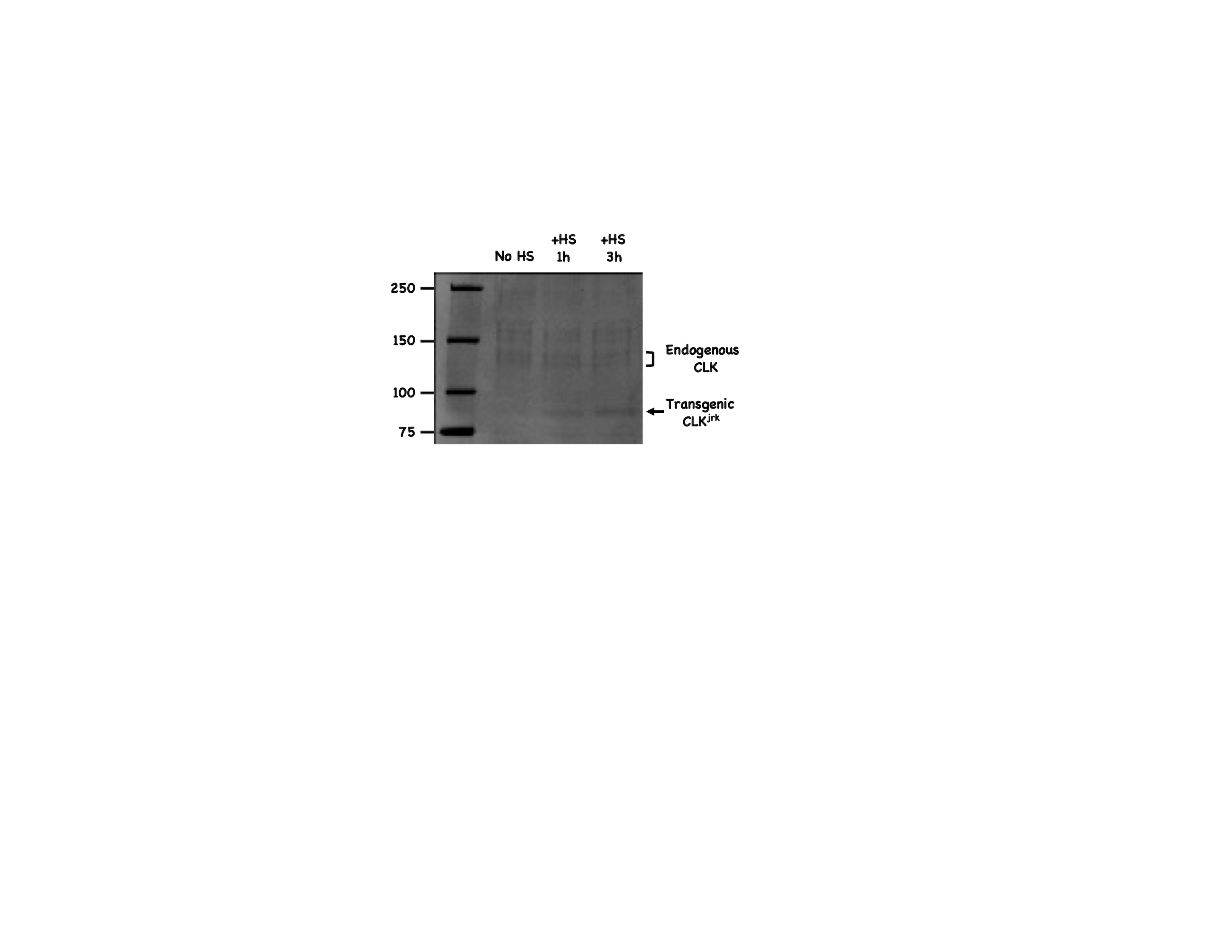

Supplement: SUPPLEMENTARY FIGURE 2 — Western analysis of HS-Clkjrk induction. HS-Clkjrk transgenic flies were entrained to a 12 h light:12 h dark schedule at 20°C. Flies in groups of approximately 100 were induced, or not, in empty food vials placed into a water bath at 36°C for 30’ at ~ZT = 7. Uninduced flies were handled, transferred to vials, but not induced. After induction (or handling only), flies were returned to food vials and incubated at 20°C for 3 h, when they were collected and flash frozen in 15 ml polypropylene tubes. The tubes were shaken and pounded, and heads were isolated using a series of sieves. 50 heads were counted out (over powdered dry ice), pulverized using a dounce-like plastic pestle, and extracts made in standard 2× Laemmli SDS loading buffer. About 15 fly head-equivalents were loaded onto a 5% polyacrylamide gel, subjected to electrophoresis, processed for western analysis using a CLK-specific antibody (gp50; Houl et al., 2006, 2008) that was used at a 1:2,000 dilution. The secondary antibody (LI-COR) was a donkey anti-guinea pig IgG conjugated with a fluor and used at a 1:20,000 dilution. The mobility of the molecular weight markers (in kD) is indicated to the left of the image. Based on mobility, the endogenous CLK and transgenic CLKjrk bands are indicated. Imaging and quantitation was done on the LI-COR Odyssey system. For quantitation, background counts (from a region of the gel that did not contain protein samples) was subtracted from both the presumed CLKjrk and endogenous CLK bands. The resulting CLKjrk intensity was greater than the adjusted intensity for the endogenous CLK band at the 3 h post-induction time point. [file Image_2.TIFF]
